# A Survey of Therapeutic Effects of *Artemisia capillaris* in Liver Diseases

**DOI:** 10.1155/2015/728137

**Published:** 2015-08-20

**Authors:** Eungyeong Jang, Bum-Joon Kim, Kyung-Tae Lee, Kyung-Soo Inn, Jang-Hoon Lee

**Affiliations:** ^1^Department of Internal Medicine, College of Korean Medicine, Kyung Hee University, Seoul 02447, Republic of Korea; ^2^Department of Microbiology and Immunology, Liver Research Institute, Cancer Research Institute and SNUMRC, College of Medicine, Seoul National University, Seoul 03080, Republic of Korea; ^3^Department of Pharmaceutical Biochemistry and Department of Life and Nanopharmaceutical Science, College of Pharmacy, Kyung Hee University, Seoul 02447, Republic of Korea; ^4^Department of Pharmaceutical Science, College of Pharmacy, Kyung Hee University, Seoul 02447, Republic of Korea

## Abstract

*Artemisia capillaris* has been recognized as an herb with therapeutic efficacy in liver diseases and widely used as an alternative therapy in Asia. Numerous studies have reported the antisteatotic, antioxidant, anti-inflammatory, choleretic, antiviral, antifibrotic, and antitumor activities of *A. capillaris*. These reports support its therapeutic potential in various liver diseases such as chronic hepatitis B virus (HBV) infection, cirrhosis, and hepatocellular carcinoma. In addition, several properties of its various constituents, which provide clues to the underlying mechanisms of its therapeutic effects, have been studied. This review describes the scientific evidence supporting the therapeutic potential of *A. capillaris* and its constituents in various liver diseases.

## 1. Introduction

The liver plays a vital role in the metabolic maintenance of homeostasis and toxic excretion of endogenous and exogenous metabolites such as those associated with drugs, alcohols, viral and fungal infections, and various noxious materials [1]. Because of this important hepatic function, liver impairment can induce the development of a variety of pathological and clinical conditions such as hepatic steatosis, inflammation, fibrotic change, and tumors. Although a considerable number of conventional medicines have been used for liver dysfunction, unanticipated side effects such as hepatic or renal toxicity, intolerance to drugs, and poor treatment occur in clinical settings. These limitations have led to the search for alternative remedies among the various herbal formulas. These herbal remedies have existed for a long time and have been useful in the management of hepatic diseases, and* A. capillaris* is one of the medicinal herbs that are frequently used for liver diseases [2, 3].

*A. capillaris*, called wormwood in English or yin chen hao in Chinese, belongs to a large genus* Artemisia*, which includes approximately over 500 species and is part of the tribe Anthemideae of the Asteraceae family [4].* A. capillaris* has been widely used as an alternative medicinal herb since ancient times to improve conditions such as pyrexia, pain, hepatotoxicity, inflammation, cholestasis, and jaundice [5]. Currently, a number of constituents including *p*-hydroxyacetophenone, *β*-sitosterol, scoparone, cirsimaritin, quercetin, arcapillin, capillin, 6,7-dimethylesculetin, 6,7-dimethoxycoumarin, capillone, capillarin, 4′-methyl capillarisin, cirsilineol, cirsimaritin, and capillarisin from* A. capillaris* have been shown to have antihepatofibrotic, anti-inflammatory, choleretic, and hepatoprotective activities [6–10]. Furthermore, more diverse pharmacological properties of* A. capillaris* have been studied in areas such as lipoapoptosis, obesity, skin cancer, antibacterial activities, and atopic dermatitis, as well as liver protection [11–15].

**Figure 1:**
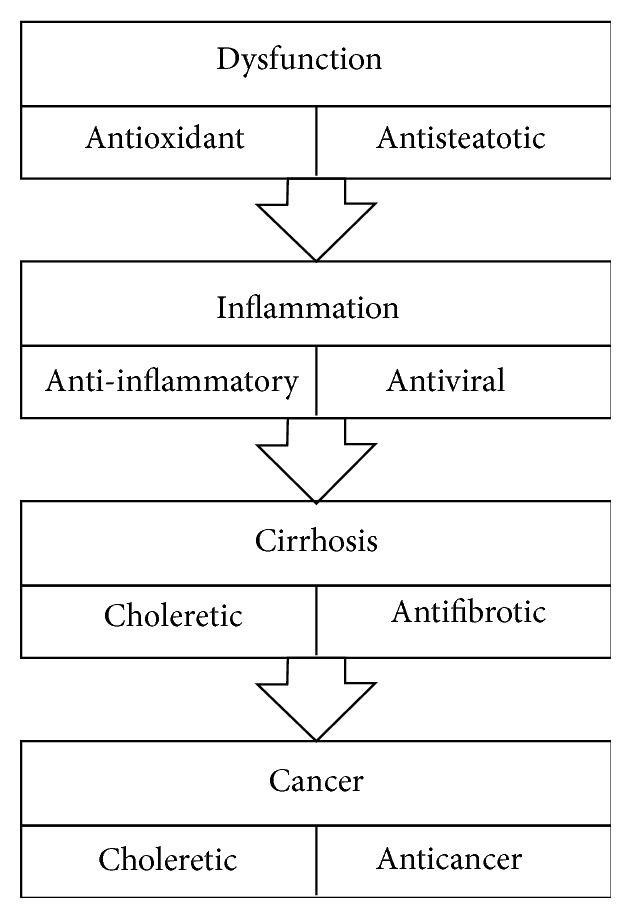
Schematic diagram of major effects of* A. capillaris* in order of progression of liver diseases. The lower box indicates reported therapeutic activities of* A. capillaris* related to liver pathological conditions in the upper box.

Despite the various medicinal applications that have been suggested for* A. capillaris* through a large number of surveys, there has been no review article that provides useful information with a well-organized collation of the pharmacological effects of* A. capillaris* and its compounds. Therefore, to meet this need, we researched a wide range of studies on therapeutic activities of* A. capillaris* and its constituents in liver diseases. In addition, we arranged these results based on the key activities that occur during the pathological developmental stages from liver dysfunction to hepatocarcinogenesis (Figure 1).

## 2. Therapeutic Effects of* A. capillaris*

For a variety of activities from* A. capillaris* relevant to liver diseases, we organized various therapeutic effects that have been reported for this plant according to each pathological step from oxidative stress to carcinogenesis (Table 1).

### 2.1. Antioxidant Effects

The liver is very susceptible to oxidative stress and plays an important role in controlling the balance between the antioxidant defense system and enhanced oxidative stress [30]. Excessive production of deleterious reactive oxygen species (ROS) could lead to liver dysfunction symptoms such as steatosis [17], alcoholic degeneration [19], cholestasis [18], and fibrosis [16] as a result of hepatocellular damage. Therefore, many studies related to the antioxidant effects of* A. capillaris* in hepatotoxicity have been conducted using chemical toxins, alcohol, fatty acids, bile duct ligation, and so forth. Oxidative injury to the liver can be confirmed by monitoring the malondialdehyde (MDA), which represents lipid peroxidative products.* A. capillaris* has been shown to significantly reduce the MDA content in the liver of rats with bile duct ligation (*p* < 0.5) [18] and 50 and 100 mg/kg of its water extract notably decreased serum levels of MDA in alcohol-pyrazole-fed rats [19].* A. capillaris* was also revealed to restore significantly reduced levels of antioxidant enzymes including superoxide dismutase (SOD), glutathione (GSH), GSH-peroxidase (Px), GSH-reductase (Rd), and catalases (CAT) under conditions of oxidative stress in* in vivo* rat models [17–19]. Furthermore, the antioxidant potential of* A. capillaris* was shown to extend to liver fibrosis [18] and hepatoprotection [16, 17, 19, 30] by observing hydroxyproline, a mark of an increase in collagen synthesis and liver function enzymes including aspartate transaminase (AST) and alanine transaminase (ALT). Therefore,* A. capillaris* is a possible candidate agent for use in the reduction of oxidative toxicities and for hepatoprotection and in liver function recovery.

### 2.2. Antisteatotic Effect

The hepatic accumulation of lipids, a characteristic feature of fatty liver disease and steatohepatitis, is strongly associated with insulin resistance [31], fibrogenic activities [32], oxidative stress [33], and lipoapoptosis [13]. Therefore, alternative therapeutic options with antisteatotic activities which can prevent oxidative and apoptotic changes in liver induced by lipids are desirable.* A. capillaris* was reported to markedly decrease the accumulated fat volume in free fatty acid-treated HepG2 cells and 3T3-L1 adipocytes [13, 14].* A. capillaris* also conspicuously improved the lipid profile including the triglyceride (TG), total cholesterol (TC), low-density lipoprotein (LDL), and high-density lipoprotein (HDL) levels in obese rats [20]. These antilipidemic activities of* A. capillaris* can be attributed to its inhibition of apoptosis via the deactivation of phosphorylated (p) c-Jun N-terminal kinases (JNK) [13] or the increase in mitochondrial *β*-oxidation [14], but more studies are needed to apply it as one of the lipid-lowering agents.

### 2.3. Anti-Inflammatory Effects

Chronic inflammatory responses in the liver are often induced by viral infection, alcohol abuse, fat accumulation, and toxic agents. Chronic inflammation causes pathological changes in liver function and, therefore, might progress to severe problems such as liver cirrhosis or cancer [34]. For this reason, controlling acute or chronic inflammation is desirable in liver disease management. Studies identifying the anti-inflammatory effect of* A. capillaris* have mainly been conducted by evaluating the levels of inflammatory cytokines. For instance, the aqueous extract of* A. capillaris* decreased the production of tumor necrosis factor- (TNF-) *α* by deactivating nuclear factor kappa-light-chain enhancer of activated B-cells (NF-*κ*B) in both lipopolysaccharide- (LPS-) and ethanol-treated HepG2 cells [21, 22].* A. capillaris* also suppressed secretion of proinflammatory cytokines such as interleukin- (IL-) 1*β*, IL-1*α*, IL-6, and IL-8 from HepG2, RAW 264.7 cells, and rat liver [6, 22, 35]. In addition, cyclooxygenase- (COX-) 2 and nitric oxide were downregulated in HepG2, RAW 264.7, RGM-1, rat insulinoma, splenocytes, and liver cells [6, 21–23, 35, 36]. These immunosuppressive effects of* A. capillaris* can be beneficial for various kinds of inflammatory conditions that occur in the liver.

### 2.4. Antiviral Effects

Viral hepatitis has the propensity to progress and possibly aggravate to liver cirrhosis or hepatocellular carcinoma. It has been revealed as a serious public health problem that affects people worldwide. Despite the available conventional therapies such as nucleoside and nucleotide analogues or interferon, these agents are fraught with challenging limitations including toxicity, drug resistance, recurrence, and unsatisfactory treatment outcomes [18, 25, 37, 38]. Traditional herbal medicines, therefore, like* Rheum palmatum*,* Scutellaria baicalensis*, and* Salvia miltiorrhiza*, have also been used for the treatment of hepatitis B virus (HBV) infections [38]. In particular, previous investigations suggested that the antiviral effects of* A. capillaris* were attributable to its isolated constituents [24, 25, 39]. Pumilaside A was among a series of compounds isolated from the 90% ethanol extract of* A. capillaris* and was discovered to have the strongest antiviral effect in HepG2.2.15 cells with a half-maximal inhibitory concentration (IC_50_) of 15.02 *μ*M and 111.3 (SI) for HBsAg, 9.0 *μ*M (IC_50_) and 185.9 (SI) for HBeAg, and 12.01 *μ*M (IC_50_) and 139.2 (SI) for HBV DNA. In addition, 19–25 compounds that inhibited viral secretions were present [24]. In addition, a recent study revealed that the compound 2f, which is a derivative of* p*-hydroxyacetophenone from* A. capillaris*, showed inhibitory effects with IC_50_ of 5.8 *μ*M and SI of 160.3 against HBV DNA replication [25]. Consequently,* A. capillaris*, which contained components with antiviral activity, was thought to be an adequate therapeutic candidate for the control of viral infections of the liver, although previous studies of this plant were mostly limited to HBV infections.

### 2.5. Choleretic Effects

Jaundice which is one of the symptoms induced by cholestasis turns skin, conjunctival membranes, and urine yellowish and is frequently seen in liver diseases such as hepatitis, liver cirrhosis, bile duct obstruction, and cancer. A number of studies of herbal therapies for hyperbilirubinemia have focused mainly on neonatal jaundice because of the adverse effects of drug treatment and exchange transfusions [40–42]. In particular,* A. capillaris* has been used for years in China, Japan, and Korea to alleviate jaundice and its choleretic effect and was officially implemented for use in Japan from 1981 to 1994 based on its various components [8, 26, 43]. Consequently, p-hydroxyacetophenone, scoparone, capillartemisin B_1_, and artepillins A and C from* A. capillaris* (dried flower, 50 mg/kg) were shown to increase bile flow without affecting the enterohepatic circulation or metabolism of bile acids in rat models. A significant increase in bile secretion of 169% was observed within 30 min of the administration of p-hydroxyacetophenone [26]. In addition, 3 mL of bile secretion for 6–8 h after treatment with the water extract of* A. capillaris* [8] might suggest the potential therapeutic benefits of* A. capillaris* on cholestatic liver dysfunctions.

### 2.6. Antifibrotic Effects

Liver fibrosis is a healing process that occurs in damaged liver tissues and can progress to liver cirrhosis or carcinoma [7, 44, 45]. The accumulation of fibrotic factors like collagen-1 and *α*-smooth muscle actin (*α*-SMA) in the liver is attributable to hepatic stellate cells induced by the tumor growth factor- (TGF-) *β* or platelet-derived growth factor (PDGF) [46]. However, conventional drugs that can block the progression of fibrogenesis or prevent liver fibrosis have not been developed yet. Concerning the antifibrotic effects of* A. capillaris*, there existed results effective against liver fibrosis in rat models. Han et al. demonstrated that 50 mg/kg dose of the water extract of* A. capillaris* had antihepatofibrotic effects in rats with bile duct ligation and regulated fibrogenic mediators such as *α*-SMA, PDGF-*β*, TGF-*β*, collagen, type I, alpha 1 (Col1A1), and tissue inhibitor of metalloproteinases TIMP1 and TIMP2 [27]. The water extract of* A. capillaris* also suppressed PDGF-*β*, TGF-*β*, and connective tissue growth factor (CTGF), which are three major profibrotic cytokines, in a carbon tetrachloride- (CCl_4_-) induced liver fibrosis rat model. However, the statistical significance was not proven, and* Artemisia iwayomogi* rather than* A. capillaris* was more effective against the fibrotic changes [3]. The *β*-sitosterol derived from* A. capillaris* administered at 120 *μ*M and 40 mg/kg significantly regulated Col1A1 and *α*-SMA in LX2 cells and dimethylnitrosamine- (DMN-) induced fibrotic mice [7]. This effect was mediated by activating caspase-3, caspase-9, and Bcl-2-associated X protein (BAX) and inhibiting B-cell lymphoma 2 (Bcl-2), myeloid cell leukemia- (Mcl-) 1, phosphorylation of mitogen-activated protein kinase (MEK), and extracellular signal-regulated kinases (ERK) [7].

### 2.7. Antitumor Effects

Primary liver cancer originating from the liver remains a serious health problem. Although the range of the geographical distribution and incidence rates are diverse with countries, gender-wise it is more prevalent in males than in females [47]. There are numerous reports on the various antitumor effects of curative treatment options like chemicals, proton, and immune therapy [48, 49]. Most studies of the inhibitory effect of* A. capillaris* against hepatoma cells have focused on apoptotic effects. Articles in Taiwan and China reported that* A. capillaris* showed growth-inhibitory effects in SMMC-7721 (human hepatoma cell line), HepG2, Huh-7, HeLa, and mouse liver cells by inducing tumor cell apoptosis [29, 50, 51]. These results are opposed to the inhibition of TGF-*β*_1_ induced apoptosis [52], but* A. capillaris* prevented apoptotic morphological changes of normal hepatocytes, not tumor cells in this study. Moreover,* A. capillaris* was also found to promote the apoptotic process in cancer cells like the human leukemia HL-60 cells or nasopharyngeal carcinoma cells (CNE-2 cells), even though they are not related to the liver [28, 53]. In view of these results, intensive studies are required to expand the possible application of the antitumor effects of* A. capillaris* to other forms of cancer.

## 3. Medicinal Effects of Various Constituents of* A. capillaris*

**Figure 2:**
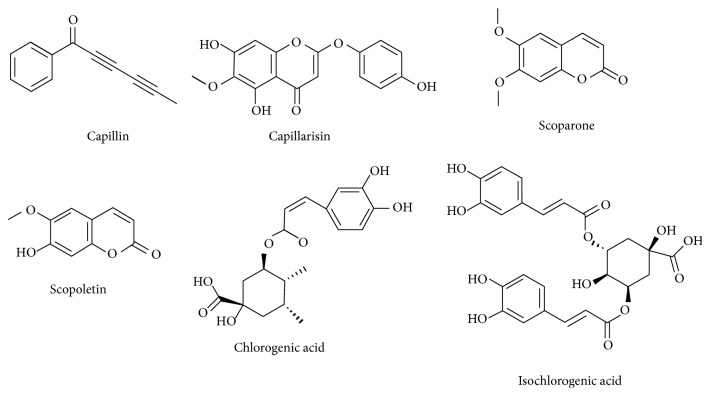
Chemical structures of selected compounds with therapeutic effects on liver diseases.

**Figure 3:**
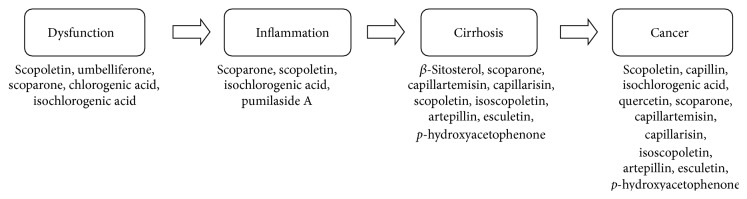
Constituents of* A. capillaris* effective to each progressive stage of liver disease.

There are a number of substances isolated from* A. capillaris*, which exhibit diverse medicinal effects applicable to different liver-related diseases (Table 2). Among those bioactive constituents, 6 compounds with remarkable medicinal effects were selected and discussed (Figures 2 and 3).

### 3.1. Capillin

Capillin, the most active and major substance found in* A. capillaris*, upregulated the apoptotic processes and thereby induced antitumor effects in human leukemia HL-60 cells by splitting DNA and activating the JNK/stress-activated protein kinases (SAPK) pathway. IC_50_ value for tumor-suppressive activity of capillin was 6.5 ± 2.9 *μ*M, which was 7.6- to 30-fold lower than those of capillin (134.9 ± 16.4 *μ*M), capillarisin (49.3 ± 12.2 *μ*M), and 6,7-dimethylesculetin (197.4 ± 17.5 *μ*M), indicating its strong growth-inhibitory activity [28]. Capillin (1–10 *μ*M) from* Artemisia monosperma* also had cytotoxic and proapoptotic effects on four human tumor cells including colon, pancreatic, lung, and larynx carcinomas [59].

### 3.2. Scoparone

Scoparone, a derivative of 6,7-dimethoxycoumarin (coumarin) isolated from* A. capillaris*, showed antioxidant properties by reducing the MDA and ALT levels in cold-reserved rat hepatocytes, thereby preventing ischemic injury induced by liver transplantation [56]. The anti-inflammatory activities of scoparone were revealed by its inhibition of the expression of IL-8, monocyte chemotactic protein-1, and NF-*κ*B subunits via I-*κ*B*α* activation in U 937 human monocytes [75]. Therefore, scoparone could be a potential candidate for the therapy of hepatitis or biliary tract infection [57].

### 3.3. Scopoletin

There are several reports of the antioxidant effects of scopoletin extracted from* Evolvulus alsinoides* and* Aegle marmelos* leaves. In addition, scopoletin from the ethyl acetate fraction of* A. capillaris* was found to have antioxidant potentials mediated by reducing the overaccumulation of ROS [17]. Scopoletin from* A. iwayomogi* also significantly promoted hepatic SOD, GSH-Px, and CAT activities in alcohol-induced obese rats [62]. In lipid metabolism, scopoletin effectively regulated hepatic lipogenic enzymes such as fatty acid synthase (FAS), acetyl-CoA carboxylase (ACC), glucose-6-phosphate dehydrogenase (G6PD), and phosphatidic acid phosphatase (PAP) and decreased TG in the liver by activating adenosine monophosphate- (AMP-) activated protein kinase (AMPK) and deactivating sterol regulatory element-binding transcription factor- (SREBP-) 1c in obese rats [62]. Scopoletin from* Erycibe obtusifolia* was mainly investigated for its suppression of IL-6 in LPS-induced RAW 264.7 cells [75] and fibroblast-like synoviocytes treated with IL-1*β* for rheumatoid arthritis [63]. The anticancer potential of scopoletin isolated from* Gelsemium sempervirens* was shown in 7,12-dimethylbenz(*α*)anthracene- (DMBA-) induced skin cancer in mice and was mediated by decreasing aryl hydrocarbon receptor (AhR), cytochrome P450 (CYP) 1A1, proliferating cell nuclear antigen (PCNA), signal transducer and activator of transcription-3 (Stat-3), survivin, matrix metalloproteinase-2 (MMP-2), cyclin D1, and c-myc mediators of carcinogenesis [65].

### 3.4. Chlorogenic Acids

Chlorogenic acid is a polyphenolic substance isolated from the methanol extracts of* A. capillaris* spectrophotometrically [68]. A previous study indicated that there was also a large amount of chlorogenic acid in the water extracts of* A. capillaris* using ultra-high-performance liquid chromatography- (UHPLC-) mass spectrometry (MS) analysis [19]. This purified compound showed an antioxidant activity comparable to *α*-tocopherol in scavenging of free radicals [68] and decreased the 2,2-diphenyl-1-picrylhydrazyl (DPPH) radical in HepG2 cells [19].

### 3.5. Isochlorogenic Acids

Isochlorogenic acids isolated from* Laggera alata* significantly inhibited the expressions of HBsAg, HBeAg, and cccDNA but not HBV DNA levels and induced heme oxygenase-1, a repressor of HBV replication, in HepG2.2.15 cells [54]. This result indicates that isochlorogenic acids may act at the translational and not transcriptional step of viral proliferation [70]. In addition, they showed anti-inflammatory effects, which were mediated by increasing antioxidant enzymes like SOD and GSH-Px [68, 71]. Furthermore, the antitumor effect of isochlorogenic acids was investigated by inducing apoptosis in murine sarcoma 180 cells via the activation of NF-*κ*B signaling and production of nitric oxide [72].

### 3.6. Capillarisin

Capillarisin (5,7-dihydroxy-2-4-hydroxyphenoxy-6-methoxychromen-4-hydroxypheno-xy), the most important chromone in* A. capillaris*, was suggested to possess a potent antitumor effect shown by its suppression of the growth of HepG2, Huh-7, and mouse liver cells. Furthermore, supercritical CO_2_ extraction of capillarisin had more remarkable effect than Soxhlet solvent in aspect of both purity value and IC_50_, which implied the importance of extraction method [51].

## 4. Conclusion

*A. capillaris* has a wide spectrum of pharmacologically effective constituents for various liver diseases ranging from fatty liver disease to liver cancer. Our findings provided at least partial and plausible explanations of the therapeutic properties of* A. capillaris* that are applicable in liver diseases. In particular, various compounds isolated from* A. capillaris* are beneficial for treating liver diseases including scoparone, scopoletin, chlorogenic acid, isochlorogenic acid, and umbelliferone. These constituents are effective for liver dysfunctions like fatty liver. In addition, scoparone, scopoletin, isochlorogenic acid, and pumilaside A are effective for hepatitis including steatohepatitis, alcoholic hepatitis, and viral hepatitis. Furthermore, scoparone, capillartemisin, capillarisin, scopoletin, isoscopoletin, artepillin, p-hydroxyacetophenone, esculetin, and *β*-sitosterol are effective for liver cirrhosis while scoparone, capillartemisin, capillarisin, capillin, scopoletin, isoscopoletin, artepillin, p-hydroxyacetophenone, esculetin, isochlorogenic acid, and quercetin are effective in liver cancer (Figure 3). In addition, Yin-Chen-Hao-Tang decoction including* A. capillaris* significantly decreased *α*-SMA, TGF-*β*_1_, and procollagen 1 by suppressing apoptosis in rats with hepatic fibrosis [76, 77]. Therefore, studies elucidating the pharmacological effects of* A. capillaris* in liver diseases are required. Hence, more studies considering diverse extraction methods, experiment techniques, efficient dosage, and related metabolism are required to verify pharmacological effects of* A. capillaris*, its constituents, and decoction in liver diseases.

## Figures and Tables

**Table 1:** Experimental results based on key activities of *Artemisia capillaris*.

Herb names (ingredients, country)	Extracts	Type	Doses	Model	Results	References
*Antioxidant effects*						
*A. capillaris* and *Picrorhiza Rhizome* 2 : 1 (Korea)	Water	*In vivo*	200 mg/kg	CCl_4_-injected rats	Liver weight↓ AST, ALT↓ Protein contents in the liver↑ Hydroxyproline↓	[16]
*A. capillaris* (Korea)	Water	*In vivo*	0.05, 0.1 g/kg	High-fat diet induced obese mice	SOD↑ GSH-px↓ CAT↓ GSH↓ TBARS↓ Carbonyl value↓ AST, ALT↓	[17]
*A. capillaris* (China)	Water	*In vivo*		Rats with bile duct ligation	MDA↓ SOD↑ GSH-Px↑ CAT↑	[18]
*A. capillaris* (Korea)	Water	*In vivo*	50, 100 mg/kg	Alcohol-pyrazole-fed rat	AST, ALT↓ ALP↓ LDH↓ ROS↓ TAC↑ GSH↑ GSH-Px↑ GSH-Rd↑ SOD↑ Catalase↑ Nrf2↑ HO1↑	[19]

*Antisteatotic effects*						
*A. capillaris* (Korea)	Ethanol	*In vitro*	100 *μ*g/mL	FFAs-treated HepG2	Lipid accumulation↓	[13]
*A. capillaris* (Korea)		*In vivo*	100 mg/kg	C57BL/6J mice fed a high-fat diet	Mitochondrial *β*-oxidation↑ Fatty acid synthase↓ Glycerol-3-phosphate Dehydrogenase↓	[14]
*A. capillaris* (scoparone, Korea)		*In vivo*		High-fat diet induced obese rats	TG↓ TC↓ LDL↓ HDL↑ SOD↑	[20]

*Anti-inflammatory effects*						
*A. capillaris* (Korea)	Water	*In vitro*		RINm5F rat insulinoma cells	NO↓ NF-*κ*B↓ Insulin release↑	[21]
*A. capillaris* (Korea)	Water	*In vitro*		LPS-induced HepG2 and rat liver	iNOS↓ COX-2↓ TNF-*α*↓ NF-*κ*B↓	[22]
*A. capillaris* (Korea)	Methanol	*In vitro*	10 *μ*g/mL	Rat splenocytes	iNOS↓ COX-2↓ TNF-*α*↓ IL-6↓	[23]

*Antiviral effects*						
*A. capillaris* (pumilaside A, China)	Ethanol	*In vitro*		HepG2.2.15	HBsAg↓ HBeAg↓ HBV DNA↓	[24]
*A. capillaris* (p-hydroxyacetophenone derivatives, China)		*In vitro*		HepG2.2.15	HBV DNA↓	[25]

*Choleretic effects*						
*A. capillaris* (Japan)	Water	*In vivo*	3 mL/day	Rats	Bile flow↑	[26]
*A. capillaris* (Japan)		*In vivo*	50 mg/kg	Rats	Bile secretion↑	[8]

*Antifibrotic effects*						
*A. capillaris* (Korea)	Water	*In vivo*	50 mg/kg	Rats with bile duct ligation	*α*-SMA↓ PDGF-*β*↓ TGF-*β*↓ Col1A1↓ TIMP1↓ TIMP2↓	[27]
*A. capillaris* (*β*-sitosterol, Korea)		*In vitro*	120 *μ*M	LX2 cells	Collagen-1↓ *α*-SMA↓ Caspases 3, 9↑ BAX↑ Bcl-2↓ Mcl-1↓ pMEK↓ pERK↓	[7]
*A. capillaris* (*β*-sitosterol, Korea)		*In vivo*	40 mg/kg	DMN-induced fibrotic mouse	Collagen-1↓ *α*-SMA↓	[7]
*A. capillaris* (China)	Water	*In vivo*		CCl_4_-induced rats	PDGF-*β*↓ TGF-*β*↓ CTGF↓	[3]

*Antitumor effects*						
*A. capillaris* (capillin, Japan)	Water	*In vitro*	2 *μ*M	HL-60 cells	Cell growth↓ JNK/SAPK activation Cytochrome c release	[28]
*A. capillaris* (China)	Water	*In vitro*	25~200 *μ*g/mL	SMMC-7721, human hepatoma cell line	Cell growth↓ G_0_/G_1_ arrest	[29]

**Table 2:** Various effects of constituents isolated and derived from *Artemisia capillaris*.

Compounds	Plants	Effects	References
Scoparone	*A. capillaris*	Vascular dilatory action	[10]
	*A. capillaris*	Choleretic	[8, 43, 54]
	*Artemisia scoparia*	Antihypertensive	[55]
	*A. capillaris*	Antioxidant	[56]
	*A. capillaris*	Anti-inflammatory	[57]

Capillartemisin	*A. capillaris*	Choleretic	[8, 58]

Capillarisin	*A. capillaris*	Antitumor	[51]

Capillin	*A. capillaris*	Anti-inflammatory	[52]
	*A. capillaris*	Antitumor	[28]
	*Artemisia monosperma*	Antitumor	[59]

Scopoletin	*A. capillaris*	Choleretic	[8]
	*Evolvulus alsinoides *L.	Antioxidant	[60]
	*Aegle marmelos *leaves	Antioxidant	[61]
	*A. capillaris*	Antioxidant	[17]
	*A. iwayomogi*	Antisteatotic	[62]
	*Erycibe obtusifolia*	Anti-inflammatory	[63, 64]
	*Gelsemium sempervirens*	Antitumor	[65]
	*Lycium barbarum*	Antitumor	[66]

Isoscopoletin	*A. capillaris*	Choleretic	[8]

Artepillin	*A. capillaris*	Choleretic	[8]

*p*-Hydroxyacetophenone	*A. capillaris*	Choleretic	[8]

Esculetin	Unknown	Choleretic	[67]

Chlorogenic	*A. capillaris*	Antioxidant	[68]
	*Phyllostachys edulis*	Antioxidant	[69]
	*A. capillaris*	Antioxidant	[19]

Isochlorogenic acid (= dicaffeoylquinic acid)	*A. capillaris*	Antioxidant	[68]
	*Laggera alata*	Antiviral and antioxidant	[70]
	*Laggera alata*	Anti-inflammatory	[71]
	*Crassocephalum crepidioides*	Antitumor	[72]

*β*-Sitosterol	*A. capillaris*	Antifibrotic	[7]

Quercetin	Chemical agents	Antitumor	[73]

Umbelliferone	Chemical agents	Antisteatotic	[74]

Pumilaside A	*A. capillaris*	Antiviral	[24]
